# 669. Metagenomic Plasma Microbial Cell Free DNA-Sequencing Assists in Diagnosis of Infections and Critical Antimicrobial Changes in Immunocompromised Hosts

**DOI:** 10.1093/ofid/ofab466.866

**Published:** 2021-12-04

**Authors:** Nicole C Vissichelli, Megan M Morales, Bharadhwaj Kolipakkam, Alexandra L Bryson, Daniel Nixon, Roy T Sabo, Amir A Toor

**Affiliations:** 1 Virginia Commonwealth University Health System, Midlothian, Virginia; 2 Virginia Commonwealth University Health, Richmond, Virginia; 3 Virginia Commonwealth University, Richmond, Virginia

## Abstract

**Background:**

Metagenomic next-generation sequencing of plasma cell-free DNA (Karius®) (plasma mcf-DNA-seq) is a noninvasive approach that may have a unique role for the diagnosis of infectious complications in immunocompromised patients. The rapid turn-around time and noninvasive nature makes this a promising supplement to standard of care.

**Methods:**

The aim of this study is to investigate the utility of plasma-mcf-DNA-seq in clinical practice; how it changes management, correlations between organism abundance over time from symptom onset and the value of negative tests. Retrospective review of plasma-mcf-DNA-seq performed, January 2020 -March 2021. Organism abundance was displayed as a heat map and graphed over time from initiation of antimicrobials. Management changes and concordance with standard of care results were compared for positive and negative tests. This study was approved by the Virginia Commonwealth University Institutional Review Board.

**Results:**

Thirty-six adult patients included: 92% immunosuppressed (11 with T cell deficits (solid organ transplant, malignancy, human immunodeficiency virus), 8 with B-cell deficits (hematologic malignancy, diabetes mellitus), and 14 with both (hematopoietic stem cell transplant, aplastic anemia)). Most tests evaluated fever (67%) and/or pneumonia (72%). Patients received a median 7 days of antimicrobials prior to testing. Twenty-one (58%) tests detected 1-5 organisms (14/21 bacteria, 8/21 fungi, and 6/21 viruses). A positive test prompted therapy changes in 14/21 patients. Of the bacterial species identified, 8/20 were considered clinically pathogenic, 3 prompted targeted treatment; 7/8 fungi identified were clinically pathogenic and resulted in antifungal therapy changes to target the species identified. Antimicrobials were de-escalated in 3 patients with negative tests. There was an exponential relationship between the abundance of pathogenic fungi over time from symptom onset, but no such relationship was seen with bacteria.

Abundance of fungi and bacteria detected on plasma mcf-DNA-seq test

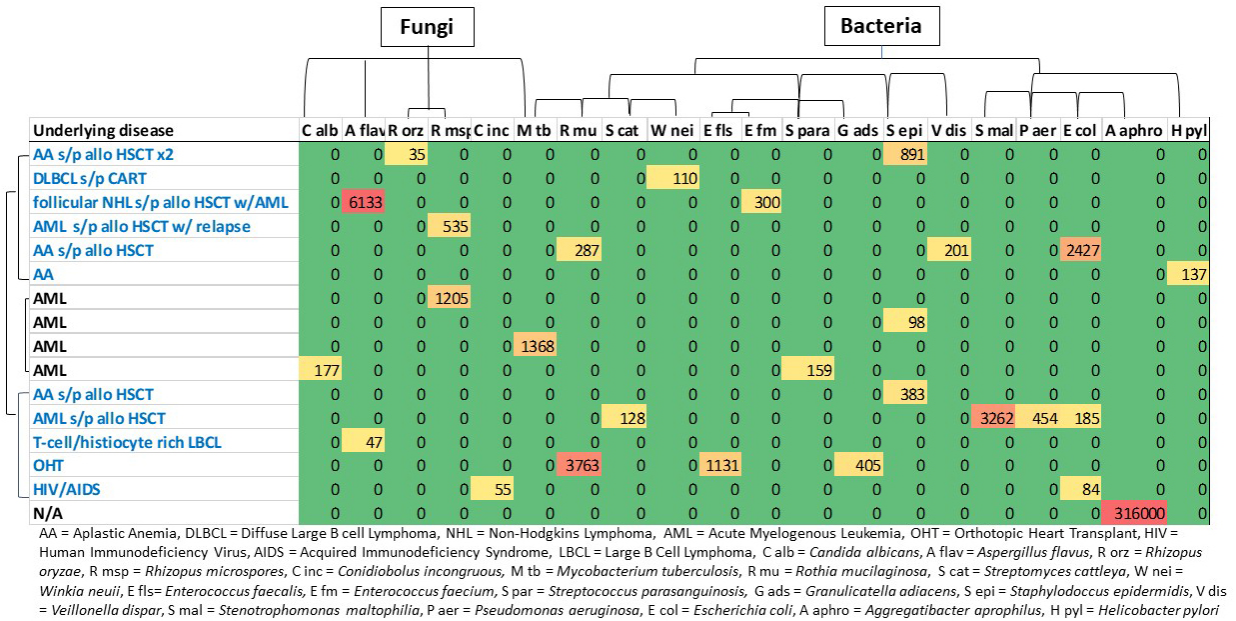

Abundance of bacteria and fungi detected on plasma mcf-DNA-seq test. Data classified by organism and level of immunosuppression. Abundance is expressed in microbial cell free DNA per microliter. Warmer colors towards red represent higher abundance.

Figure 1. Bacteria abundance from date of symptom onset.

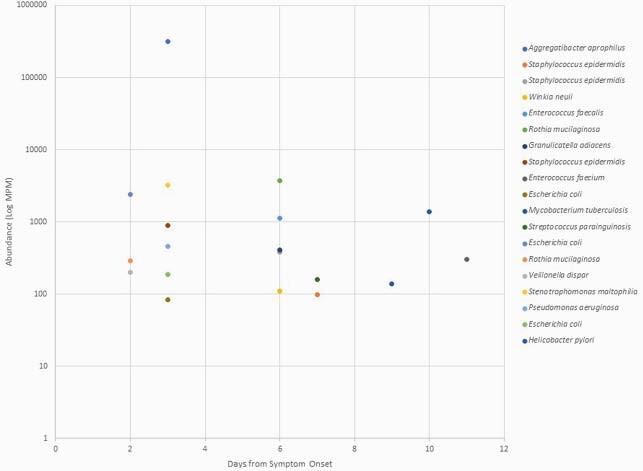

There was no clear trend in bacterial abundance over time from symptom onset. Most bacteria detected were not considered clinically pathogenic.

Figure 2. Fungi abundance from date of symptom onset

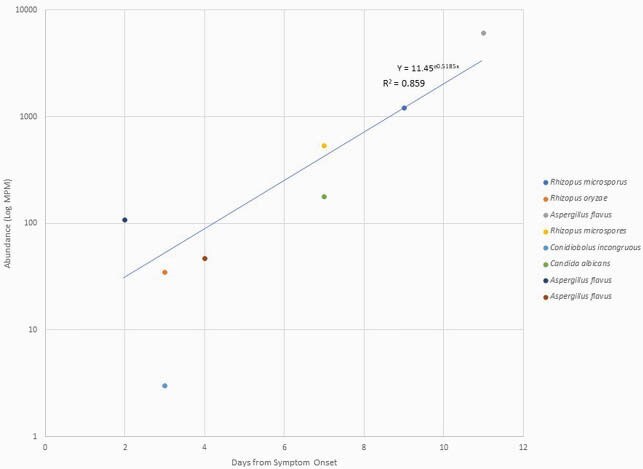

There was an increasing trend in the abundance of fungi detected from time of symptom onset. Seven of the 8 fungi detected were considered clinically pathogenic.

**Conclusion:**

Plasma-mcf-DNA assisted in making critical management changes including initiation of treatment for identified organisms and de-escalation of antimicrobials. Plasma-mcf-DNA is a promising approach for a non-invasive rapid diagnosis.

**Disclosures:**

**All Authors**: No reported disclosures

